# An Unusual Case of Peritoneovaginal Fistula With Fimbrial Prolapse in a Post-hysterectomy Woman

**DOI:** 10.7759/cureus.46096

**Published:** 2023-09-27

**Authors:** Ajay Halder, Gargi Gautam, Khushbu Dhubey, Shraddha KS

**Affiliations:** 1 Obstetrics and Gynaecology, All India Institute of Medical Sciences, Bhopal, Bhopal, IND

**Keywords:** genitourinary fistula, invasive diagnostic technique, post hysterectomy woman, chronic vaginal discharge, prolapsed fimbrial end, peritoneovaginal fistula

## Abstract

A fairly uncommon differential diagnosis for chronic vaginal discharge and sexual dysfunction in post-hysterectomy women is peritoneo-vaginal fistula. It can present with or without fimbrial end prolapse. It is also a rare differential of genito urinary fistula due to its comparable presentation in post-hysterectomy women. Patients' morbidity and a number of unneeded conservative treatments, including the use of antibiotics and superfluous tests, can be brought on by surgeons' uncertainties related to its diagnosis. Prophylactic salpingectomy and proper surgical technique are essential for avoiding these problems as summarised from previous literature. Hereby, we are presenting a case of a 30-year-old woman who underwent abdominal hysterectomy and has been complaining of persistent watery vaginal discharge for a year after the procedure. As genito-urinary fistula is a common relative differential of the presenting case scenario, hence workup was done to rule it out but there were no noteworthy discoveries found from the workup. Due to the diagnostic conundrum, we scheduled the patient for a diagnostic laparoscopy that was followed by the detection of the peritoneo-vaginal fistulous tract with prolapsed fimbrial end, repair of tract followed by bilateral salpingectomy.

## Introduction

Fallopian tube prolapse (FTP) following hysterectomy was first noted by Pozzi in 1902 [1]. Approximately 0.01-0.05% of all hysterectomies result in FTP entering the vaginal vault, making it a very uncommon event [1]. The most frequent differential diagnosis for persistent vaginal leakage following a hysterectomy is a vesicovaginal fistula [2]. A very uncommon post-operative complication of gynaecological surgery is a peritoneo-vaginal fistula causing persistent vaginal discharge which has been connected to prolapsed fimbria in some cases. An erroneous identification of granulation tissue at the vault that causes confusion with recurrent cervical cancer and vesico-vaginal fistulas can delay diagnosis and, in certain situations, have potentially lethal effects including peritonitis. There have only been a few such cases tracked till now and this condition is also excluded from contemporary gynaecology textbooks based on its rarity. Because partial salpingectomy might produce a recurrence of vaginal discharge and further tension on the tubal remnant can cause chronic discomfort and dyspareunia, total salpingectomy with closure of the vault defect is seen to be the best course of treatment [3].

## Case presentation

**Figure 1:**
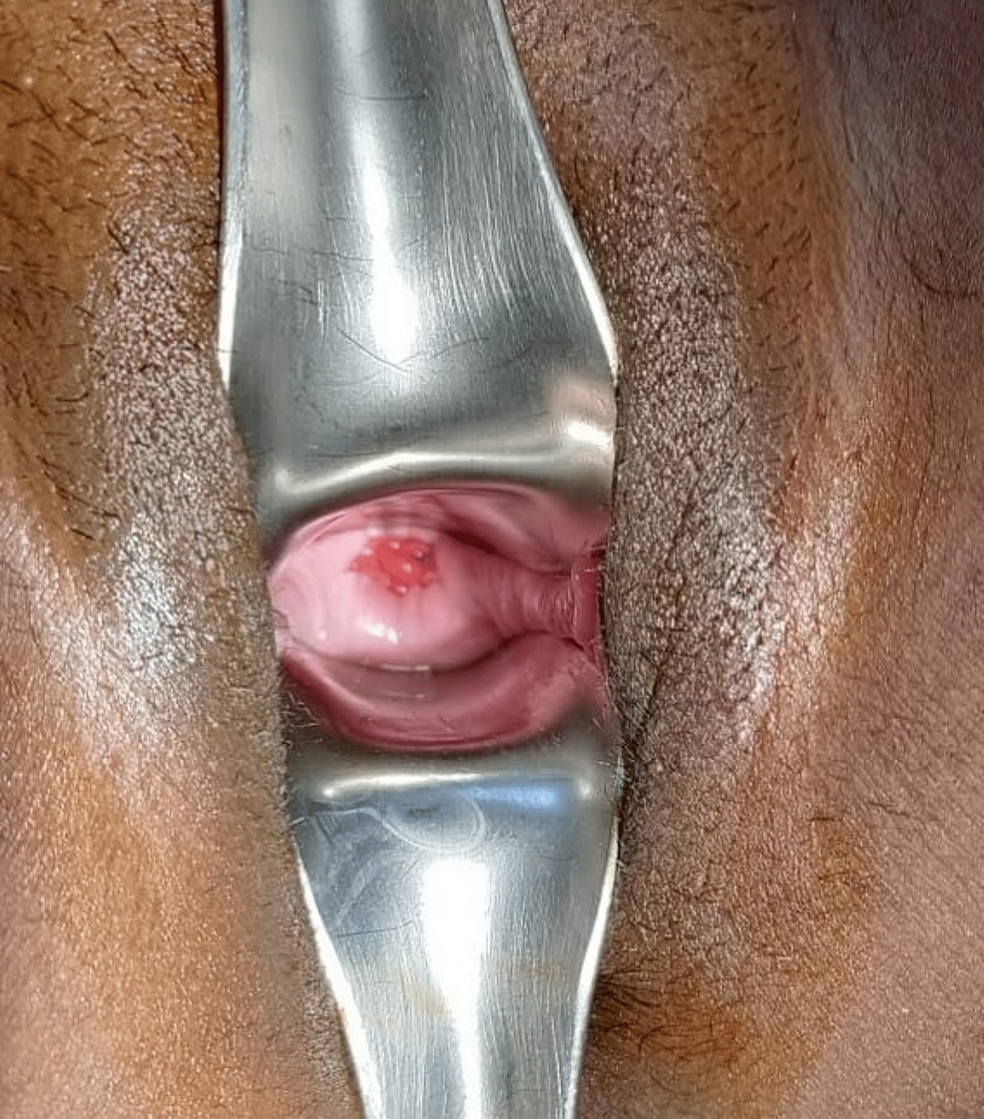
Prolapsed fallopian tubes that resemble granulation on the vaginal vault

**Figure 2:**
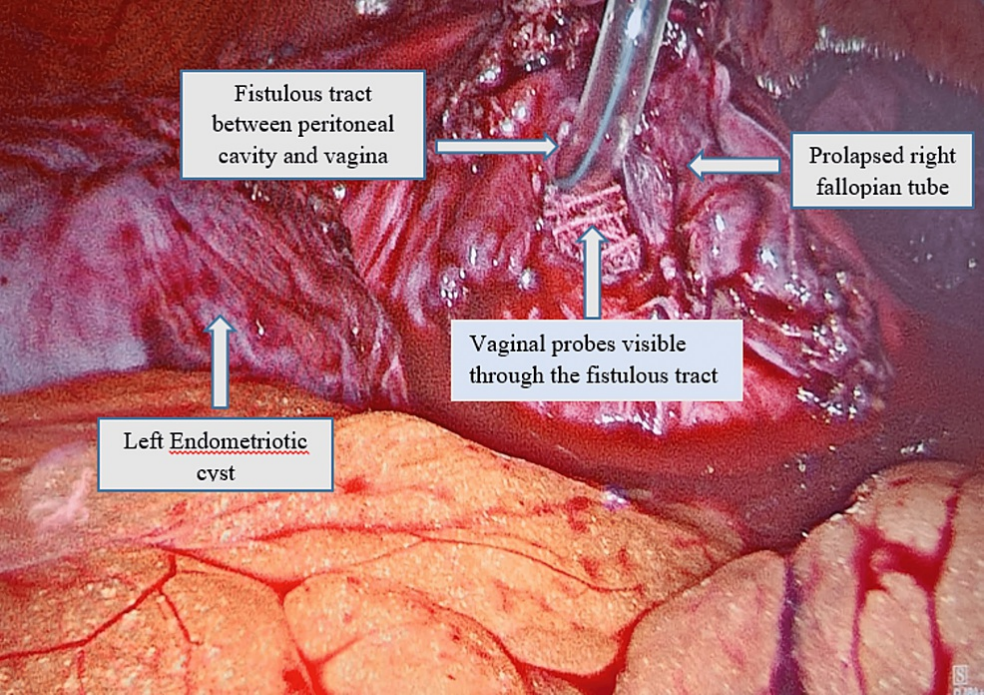
Laparoscopic findings showing prolapsed fimbrial end from peritoneo-vaginal fistula

A 30-year-old gravida 3, para 3 (P3L3), all vaginal deliveries, post-abdominal hysterectomy that was done two years ago because of abnormal uterine bleeding with fibroid uterus, was refractory to medical management. However, details of intraoperative findings were not available but no notable event explained by the patient in the postoperative period. She presented to us with chief complaints of continued watery vaginal discharge that increases during sexual intercourse and activities causing raised intra-abdominal pressure since the last year post-hysterectomy. She was requiring a sanitary napkin to maintain perineal hygiene and previously had taken an antibiotic course prescribed outside. There was no notable finding in the general examination. On pelvic examination, blood-tinged odourless discharge was noted with a red friable fleshy mass resembling granulation tissue of around 1cm seen at the vaginal cuff, as shown in Figure 1. On culture sensitivity, no significant findings were noted. A small defect was felt at the right angle of the vaginal vault; however, the cough stress test was negative. Triple swab test was also done using methylene blue dye and oral pyridium during the examination and was found to be negative. In ultrasonography, no significant findings were noted, and in CT urography, there was no contrast leak found. A decision was taken to proceed further with invasive diagnostic techniques, and she was posted for diagnostic laparoscopy and proceeded in the same setting. Intraoperative, vaginoscopy showed the same granulation tissue mass that was resected under vision, a defect was noted at the excised site post resection. A nasogastric (NG) tube was inserted through the defect. Laparoscopy camera ports were inserted, and findings were noted as shown in Figure 2. The pouch of Douglas was obliterated with dense adhesion between bowel loops and adjacent tissue enveloping bilateral fallopian tubes inside the adhered mass. Further laparoscopic dissection and exploration revealed the same NG tube that was used as a probing object from the vaginal end, with bilateral fallopian tubes prolapsed and attached to the vaginal cuff. Under the guidance of an inserted NG tube and folded gauze (Figure 2), a fistulous tract was delineated and repaired, followed by a bilateral salpingectomy in the same setting. The postoperative period was uneventful, and complaints were resolved post-procedure. The histopathology of the resected tissue was suggestive of fibro-collagenous tissue bits focally lined by simple coloumnar epithelium, with subepithelial cells showing dense lymphoplasma admixed with eosinophils 

## Discussion

An inappropriate connection between the vaginal cuff and the fallopian tube leads to prolapsed fallopian tubes and salpingovaginal fistulas. Tubes can occasionally prolapse when they are secured close to the apex of the vaginal cuff after a hysterectomy [4]. Fallopian tube prolapse and peritoneovaginal fistula have multiple potential causes. Predisposing variables may include malnutrition, post-radiation treatment, cancer, poorly managed diabetes mellitus, poor wound healing brought on by prolonged steroids, and others [5]. Postoperative hematoma, infection, insufficient preoperative vaginal preparation, challenging surgery, and elevated intraabdominal pressure are additional risk factors [5]. Early prolapses are frequently brought on by intense physical activity or early sexual engagement, which weakens the vaginal vault scar as could be a potential cause in presented age group patients [6]. The condition's rarity can be attributed to the fact that the tubal end would only be more likely to herniate if it were sufficiently lengthy and flexible [7]. Peritoneo-vaginal fistula with prolapsed fimbrial end of fallopian tube can mimic genitourinary fistula due to its similar presentation and misleading symptoms and it is challenging to differentiate and diagnose by examination and radiological diagnostic methods. In the presented case, nasogastric tube of 12-French was introduced into the peritoneovaginal fistula via the vaginal route after resecting prolapsed tissues under vaginoscopy to distend the fistulous tract. This technique verified the diagnosis, helped us to securely separate the fallopian tube from the vagina and bladder, and improved the salpingectomy's dissecting planes by applying traction more effectively. The probes also enabled manipulation of the fallopian tube, allowing for better distendment of the tract by using folded gauze pieces and NG tube, not any expensive, rarely available instrument. A prolapsed fallopian tube has the traditional columnar cells and cilia as well as sheets of cells as found in the presented case [4].

## Conclusions

This case demonstrates a step-by-step examination process for vaginal leakage, potentially requiring diagnostic laparoscopy if no other explanations are found. Fallopian tube-vaginal fistulas can be treated laparoscopically or vaginally to minimize invasiveness. Due to the rarity of such case scenarios and surgeons' minimal exposure to the same, patients are forced into a cycle of unneeded testing for illnesses that can present with neogrowth on vault mimicking carcinoma of cervix and genitourinary fistula. Differential diagnosis of persistent vaginal discharge should include peritoneovaginal fistula in post-hysterectomy women considering increasing instances for the same. To prevent fallopian tube prolapse, systematic salpingectomies may be helpful following hysterectomies for benign gynecological disorders. It would also prevent fallopian tube prolapse in addition to ovarian cancer.
